# Role of *Groucho* and *Groucho1-like* in Regulating Metamorphosis and Ovary Development in *Nilaparvata lugens* (Stål)

**DOI:** 10.3390/ijms23031197

**Published:** 2022-01-21

**Authors:** Han Gao, Xiaojuan Jiang, Shiwen Zheng, Yan Li, Xinda Lin

**Affiliations:** 1 College of Life Sciences, China Jiliang University, Hangzhou 310018, China; 15255296862@163.com (H.G.); Jiangxiaojuanmm@163.com (X.J.); zzzzssswww@163.com (S.Z.); 15825533072@163.com (Y.L.); 2 College of Biotechnology and Bioengineering, Zhejiang University of Technology, Hangzhou 310014, China

**Keywords:** *Nilaparvata lugens*, juvenile hormone, Ecdysone, NlGroucho, *NlGroucho1-like*

## Abstract

Juvenile hormone and ecdysone are key regulators in the metamorphosis and development. Grocho (Gro) is a highly conserved protein required for metamorphosis and development. Brown planthopper (*Nilaparvata lugens*) is a major pest affecting rice production in China and many Asian countries. Although the molecular function of *Gro* has been investigated in holometabolous insects such as *Aedes aegypti* and *Drosophila melanogaster*, their role in the hemimetabolous insect, brown planthopper, and the relationship between *NlGro*/*NlGro1-L* and JH/ecdysone signaling pathway, remained unknown. In this study, *NlGroucho* (*NlGro*) and *NlGroucho1-like* (*NlGro1-L*) were cloned. An analysis of the predicted protein sequence showed that NlGro has highly conserved Q domain and WD40 domain, and NlGro1-L has a highly conserved WD40 domain. The expression profiles of both genes were studied by quantitative real-time PCR (qRT-PCR). Their relative expressions were high in egg, head, wing, ovary, and testis. *NlGro* and *NlGro1-L* were found to interact genetically with juvenile hormone and ecdysone signaling by hormone treatment and RNAi of JH/ecdysone signaling-related genes. Moreover, when *NlGro* or *NlGro1-L* was down-regulated alone, the survival rate was decreased, the ovarian development was delayed, and the oviposition was also affected. All defects were aggravated when *NlGro* and *NlGro1-L* were down-regulated together. This study will help to develop new pesticides on the basis of the function of *NlGro* and *NlGro1-L*, and provide new possibilities for the control of *Nilaparvata lugens*.

## 1. Introduction

Insects must undergo a process of molting and metamorphosis during their growth and development. The main regulators of insect metamorphosis are juvenile hormone (JH) and ecdysone (or 20-hydroxy-ecdysone, ED) [1,2]. Juvenile Hormone (JH) is a sesquiterpene compound and was first discovered by Sir Wigglesworth in the *Rhodnius prolixus* [3]. Juvenile hormone controls the basic physiological functions of insect development, reproduction, pheromone production, and antennae formation [4]. Increasing or decreasing the level of juvenile hormone will affect ovarian development [1,2]. Ecdysone is a sterol compound that is synthesized by insect prothoracic gland and secreted into the hemolymph [5]. Both JH and ED are key regulatory hormones in the metamorphosis and development [6,7,8]. Moreover, juvenile hormone signaling regulates ovarian development and maturation, partly through the interaction with ecdysone signaling [6,7,8,9]. Juvenile hormone also regulates the expression of vitellogenin in fat body and hemolymph [1,2].

Thus far, the *Groucho* (*Gro*) gene family is only found in metazoans, and is named after the first identified *Drosophila* mutant phenotype [10]. *Drosophila Gro* is widely expressed throughout the development [11]. The homologous gene Transducin-Like Enhancer of split (TLE) in medaka fish is mainly expressed during embryo and ear development [12]. The human Gro homologue has high structural similarity with β-transducin and is close to *Drosophila* Gro (a member of the Enhancer of split (E(spl)) complex), so it is named the TLE protein [13,14,15]. The homologous *Gro*-related gene *Grg* protein was also isolated from mice [16]. The homologue of *Caenorhabditis elegans* is named Unc-37 (ESG) protein and its 612 amino acid sequence protein is the smallest known Gro protein [16,17]. The *Drosophila* embryonic development requires maternal Gro protein, and post-embryonic development depends on the expression of zygotic Gro [11]. The primary structure of Gro has five domains, which are defined by its evolutionary conservation, namely Q, GP, CcN, SP, and WD. The most highly conserved and strictly characterized domains of the Gro family are the amino terminal Q domain and the carboxy terminal WD repeat domain [16]. The other three domains, GP, CcN, and SP, are the middle variable domains [16]. Despite the different nomenclature in different species, Gro still shows high conservation, especially in the carboxy terminal WD domain, most of which have at least 80% amino acid identity [16]. Gro plays an important role in embryonic development, neurogenesis, and sex determination [17,18,19,20].

The *Drosophila Gro* interacts genetically with the E(spl) complex [21]. Gro does not directly bind to DNA, but it can bind to transcription factors to regulate transcription [21]. Both *Drosophila* Gro and human TLE1 proteins have been shown to physically interact with Hairy (human homologue HES) [18]. The Trp-Arg-Pro-Trp (WRPW) motif found at the carboxyl terminal of Hairy is necessary and sufficient for interaction with Gro [18]. Furthermore, this interaction between the WRPW motif and Gro is necessary for transcriptional repression [16,22]. The *Drosophila* Gro also interacts with Hairy and Deadpan regulatory proteins in segmentation and sex determination [23,24]. *Aedes aegypti* Hairy is a downstream gene of Met, and Gro directly interacts with Hairy’s conserved C-terminal tetrapeptide WPRW to regulate the signal transduction of JH-Met [25]. There are short Grg/TLE family members in both humans and mice; these short members only include a Q domain responsible for the dimerization of Grg/TLEs and a glycine-proline-rich GP domain to repress transcription [16,22,26].

The brown planthopper (*Nilaparvata lugens* Stål) is a monophagous pest that can only feed and reproduce on rice and common wild rice. The brown planthopper has a migratory habit and is a major threat to rice production in China and many other Asian countries. Although the molecular function of *Gro* has been investigated in holometabolous insects, such as *Aedes aegypti* and *Drosophila melanogaster*, their role in the hemimetabolous insect, brown planthopper, and the relationship between *NlGro*/*NlGro1-L* and the JH/ecdysone signaling pathway, especially key players of the JH/ecdysone signaling pathway, such as E93, remain unknown. In this study, *NlGro* and *NlGro1-L* were cloned, and their expression at different developmental stages and in different tissues was measured; the relationship between *NlGro*/*NlGro1-L* and JH/ecdysone signaling pathway was also investigated through survival rate, development, and reproduction.

## 2. Materials and Methods

### 2.1. Insect Culture

The experimental insects were reared in our laboratory with rice of the IIyou023 variety. The rice was cultured with nutrient solution at 25 °C. The relative humidity is around 65%, and the photoperiod is 14L:10D (h). The nutrient solution used was changed daily. The nutrient solution contained NH_4_NO_3_ (114.3 mg/L), NaH_2_PO_4_•2H_2_O (50.4 mg/L), K_2_SO_4_ (89.3 mg/L), MgSO_4_•7H_2_O (405 mg/L), CaCl_2_ (110.8 mg/L), and trace elements (Sangon Biotech, Shanghai, China) [27].

### 2.2. RNA Extraction, cDNA Synthesis, and qRT-PCR

Brown planthopper individuals of every stage of development were collected over a 3-day period for total RNA extraction, including 40 first instar nymphs, 20–30 of each of second, third, fourth, and fifth instar nymphs, 15 brachypterous 7-day old adults, and 15 macropterous 7-day old adults. Forty embryos were collected on each day of the 9-day embryonic development stage. Three biological replicates were used. Total RNA was extracted with RNAiso Plus (TaKaRa, Dalian, China). The precipitate was dissolved in DEPC-treated water, and then the concentration and purity were measured with a micro-spectrophotometer (Allsheng, Hangzhou, China). The Transcriptor First Strand cDNA Synthesis Kit (Roche, Shanghai, China) was used for synthesis of cDNA. A 20 μL reaction was used: 1 μg total RNA, 1 μL Oligo dT primer, water added to 13 μL, and incubated in a water bath at 65 °C for 10 min, then 4 μL buffer was added, 2 μL dNTP, 0.5 μL inhibitor, and 0.5 μL Transcriptase, in a water bath at 55 °C for 30 min, finally transferred to a water bath at 85 °C for 5 min.

Templates for quantitative real-time PCR (qRT-PCR) were prepared by collecting insects at different embryonic development stages: females laid eggs on rice seedlings after mating, and 1, 2, 3, 4, 5, 6, 7, 8, and 9 days after the eggs hatched. The insects were also collected at different developmental stages: egg, first, second, third, fourth, and fifth instar nymph, as well as short-winged females, long-winged females, short-winged males, and long-winged males. Templates for the qRT-PCR of different tissues: head, thorax, forewing, leg, mid-gut, ovary, or testis. The mid-gut is a tube with the same thickness. The mid-gut was dissected based on its relative position between the fore-gut and the malpighian tube/hind-gut. To further study the correlation between *NlGro/NlGro1-L* and JH/ED signaling, we also measured the expression of *NlGro* and *NlGro1-L* after down-regulating hormone-related genes (*NlMet*, *NlβFtz-f1*, *NlTai*, *NlKr-h1*, *NlE93*, *NlECR*, *NlUspA*, *NlUspB*, and *NlBr*) by RNAi.

Primer3 was used to design primers for qRT-PCR. The primer sequences were listed in Table S1. Genes tested by qRT-PCR: *NlGro*, *NlGro1-L*, *NlMet*, *NlβFtz-f1*, *NlTai*, *NlKr-h1*, *NlE93*, *NlECR*, *NlUspA*, *NlUspB*, *NlBr*, and *NlVg*. For measuring the transcriptional response to JH/ED, *NlActin* was used as a reference gene. For the rest of the experiments, *NlRP15* was used as a reference gene. The reference genes were selected based on a previous study [28]. Moreover, we carried out a qRT-PCR experiment to compare the stability of the reference genes (*NlRP15* and *NlActin*), by which we selected a relatively stable reference gene for different experiments (Figure S1). The primers of qRT-PCR were shown in Figure S2. qRT-PCR was performed using the YESEN Hieff qRT-PCR SYBR Green Master Mix kit (*actin* was used as the reference gene). The reaction system was SYBR Green Master Mix (1×) 10 μL, cDNA template 2 μL, upstream and downstream primers 0.4 μL each (10 µM), and up to 20 μL with DEPC water. Three biological replicates were used for each sample. ABI StepOnePlus quantitative real-time thermocycler was used. Two-step amplification was used. The first step (pre-denaturation): 95 °C, 2 min, and one cycle; the second step (PCR reaction): first 95 °C 10 s and then 60 °C, 30 s, 40 cycles. The data were analyzed with the 2^−ΔΔCt^ method [29].

### 2.3. Cloning

The template used to clone *NlGro* and *NlGro1-L* was prepared from nymphs. cDNA was synthesized using Transcriptor First Strand cDNA Synthesis Kit (Roche, Shanghai, China). Primers were designed using Primer3 (http://primer3.ut.ee/, accessed on 15 September 2017) based on the sequences downloaded from the NCBI (*NlGro*: 111053289; *NlGro1-L*: 111047810). The primer sequences were shown in Table S2. The cloning vector pMD18-T (TaKaRa) was used, and the plasmid was sent for sequencing after cloning (Sangon Biotech Co. Ltd., Shanghai, China).

### 2.4. JHIII and Ecdysone Treatment

Juvenile hormone III(JH III) and Hydroxyecdysone (ED) were purchased from Sigma (Sigma-Aldrich, Shanghai, China). Brown planthoppers at the fifth-instar nymph stage (0–12 h) were collected and treated with 0.2 μL JH (1 μg/μL) or 0.2 μL ED (0.05/0.1 μg/μL). The same amount of acetone was used as the control. A 5 μL micro-injection needle (Hamilton 700, Hamilton Laboratory Equipment Co., LTD, Shanghai, China) was used to topically apply 1 µL of the prepared hormone solution or acetone (control) on the back of the fifth instar nymph. At least 50 nymphs were treated for each sample. After the recovery of the treatment, the insects were cultured in a glass tube containing rice seedlings. Samples of 12 nymphs (three replicates in total) were collected and total RNA was isolated. After 6 h, 12 h, and 24 h, samples were taken, total RNA was extracted, and cDNA was synthesized for qRT-PCR.

### 2.5. dsRNA Synthesis and RNAi

A PCR product with a size about 500 bp was used to synthesize double-stranded RNA (dsRNA). The primers were shown in Table S3. Region of dsRNA and qRT-PCR primers was shown in Figure S2. GFP dsRNA was used as a control. dsRNA was synthesized according to the instructions of the T7 RiboMAX Express RNAi System kit (Promega, Madison, WI, USA), and frozen at −20 °C for later use.

The nymphs were injected with 0.2 μg dsRNA of the corresponding gene using the Narishige Injection System (MN-151, Narishige, Japan). The control group was injected with the same amount of *GFP* dsRNA. After injection, the brown planthoppers were allowed to recover for 2 h and then cultured on rice seedlings. The brown planthoppers were cultured in an incubator in groups of 10. After RNA extraction, cDNA was synthesized and qRT-PCR was used to measure the expression level of genes. The results were analyzed using the 2^−ΔΔCt^ relative expression method [29]. In addition, the survival rate and molting rate were calculated.

### 2.6. Ovary Dissection, Grading, and Oviposition

The nymphs of *Nilaparvata lugens* were dissected 3 days after emergence, and used for ovarian grading. The ovaries of females were dissected and graded. The grading standard can be referred to our previous report [6]. The dissected ovaries were fixed with 4% paraformaldehyde (Sangon) for 20 min. Then, we rinsed them with PBS three times, removed the fixed ovaries, placed them on a glass slide, added an appropriate amount of glycerin, and took photos with a microscope (eclipse 80i, Nikon, Japan).

The fifth instar nymphs injected with dsRNA were cultured. Males were added to mate with the females on the day of emergence. Each treatment guarantees 20 individuals. Three biological replicates were used. We recorded the time of the eggs laid and the number of eggs each day for calculating the pre-oviposition period, as well as the total number of eggs laid until the female died.

### 2.7. Western Blot

The fifth instar nymphs injected with dsRNA were dissected and sampled 3 days after emergence, while the sample was ground with a grinding pestle on ice and centrifuged to collect the supernatant. The protein samples were subjected to SDS-PAGE electrophoresis (Sangon), and transferred to PVDF membrane (ThermoFisher Scientific, Shanghai, China) after 150 min. We added an appropriate amount of 5% non-fat milk and blocked it slowly in a shaker. Then, it washed with 1X PBST, primary antibody anti-Vg and ACTB antibody was added (Rabbit anti-Vg antibody is a gift from Zhou Qiang of Sun Yat-Sen University, 1:10,000 dilution; Mouse anti-ACTB antibody, purchased from Sangon, 1:1000 dilution), and then incubated for 1 h. We washed it with 1X PBST and incubated it with HRP-labeled secondary antibody (Goat anti-rabbit or horse anti-mouse IgG-HRP, Cell Signalin Technology, Shanghai, China, 1:5000 dilution), and then washed it with 1X PBST.

## 3. Results

### 3.1. Sequence Analysis of NlGro and NlGro1-L

**Figure 1:**
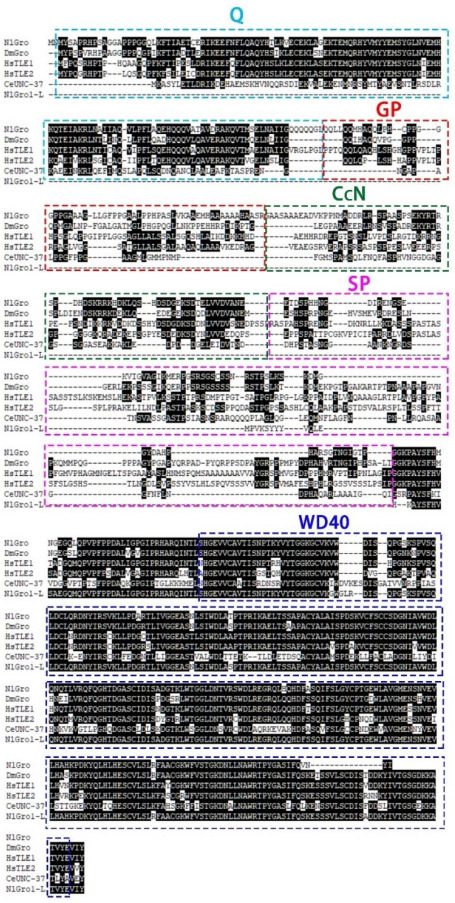
Alignment of NlGro and NlGro1-L.

*NlGro* and *NlGro1-L* were cloned and the amino acid sequences of NlGro and NlGro1-L were predicted and compared with the homologs of Gro family proteins from other species. The Gro family protein contains five conserved functional domains, among which the Q domain at the amino terminal and the WD40 domain at the carboxy terminal are highly conserved. The Q domain can not only inhibit basic transcription, but also activate transcription and act as a dimerization domain. The WD40 domain can be used for the interaction with other proteins. NlGro also contains highly conserved Q domain and WD40 domain (Figure 1). Although NlGro1-L does not contain Q domain, it has a WD40 domain (Figure 1). The phylogenetic analysis suggests that the brown planthopper is more closely related to *Frankliniella occidentalis*, *Onthophagus taurus*, *Drosophila melanogaster*, *Bemisia tabaci*, *Riptortus pedestris*, and *Halyomorpha halys* (Figure S3).

The blue dashed box represents the conserved Q domain, and the red dashed box represents the highly conserved WD40 domain.

The neighbor-joining (NJ) method was used to construct a phylogenetic tree of Gro homologs of different species. NlGro and its homologues in *Frankliniella occidentalis*, *Onthophagus taurus*, and *Drosophila melanogaster*, have high homology, while NlGro1-L and its homologues in *Bemisia tabaci*, *Riptortus pedestris*, and *Halyomorpha halys* have high homology. *Caenorhabditis elegans* seems to be the ancestor of the Gro family. *NlGro*, *Nilaparvata lugens Gro* (XP_022195835.1); *DmGro*, *Drosophila melanogaster Gro* (NP_733133.1); *HsTLE1*, *Homo sapiens TLE1* (NP_001290032.1); *HsTLE2*, *Homo sapiens TLE2* (NP_003251.2); *CeUNC-37*, *Caenorhabditis elegans UNC-37* (NP_491932.1); *NlGro1-L*, *Nilaparvata lugens Gro1-L* (XP_022189353.1).

### 3.2. Expression Profiles of NlGro and NlGro1-L

**Figure 2:**
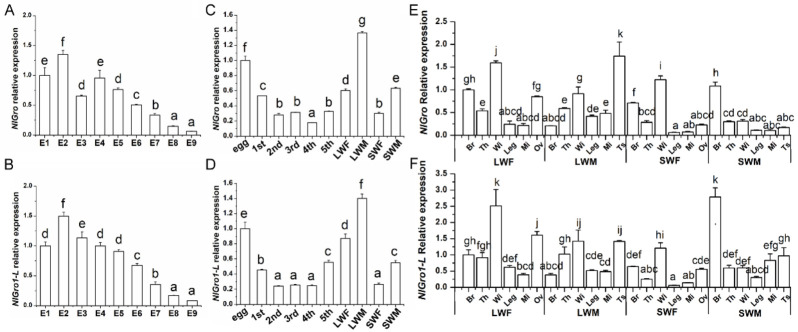
Expression profiles of *NlGro* and *NlGro1-L.* (**A**) Expression of *NlGro* at different embryonic stages. (**B**) Expression of *NlGro1-L* at different embryonic stages. (**E**) Embryo; 1–9 day(s) after eggs were laid. (**C**) Expression of *NlGro* at different developmental stages. (**D**) Expression of *NlGro1-L* at different developmental stages. Egg: egg; 1st to 5th: first to fifth instar nymph; LWF: long-winged female, LWM: long-winged male, SWF: short-winged female, SWM: short-winged male. (**E**): Expression of *NlGro1* in different tissues. (**F**) expression of *NlGro1-L* in different tissues. Br: Brain; Th: Thorax; Wi: forewing; Leg: Leg; Mi: mid-gut; Ov: Ovary; Ts: testis; LWF: long-winged female, LWM: long-winged male, SWF: short-winged female, SWM: short-winged male. One-way analysis of variance (Duncan’s method) was used. For the letters a–h, the same letter indicates that there is no significant difference, and different letters indicate a significant difference between the two; the farther the letter is, the greater the difference between the two, *p* < 0.05.

The expression levels of *NlGro* and *NlGro1-L* at different developmental stages and in different tissues were detected by qRT-PCR. The results showed that the expression profiles of *NlGro* and *NlGro1-L* in different embryonic stages were similar (Figure 2A,B). The expression of *NlGro* and *NlGro1-L* tends to decrease from different embryonic stages to different nymph stages (Figure 2A–D). The expression profiles of *NlGro* and *NlGro1-L* at different developmental stages are similar (Figure 2C,D). The expression of *NlGro* and *NlGro1-L* in the long-winged adults is higher than that in the short wings (Figure 2C,D). The expression profiles of *NlGro* and *NlGro1-L* in different tissues are also similar (Figure 2E,F). In summary, the expressions of *NlGro* and *NlGro1-L* are relative higher in the heads, wings, and ovaries of the females. The expressions in ovary and testis are higher, indicating that *NlGro* and *NlGro1-L* may play a role in reproduction.

### 3.3. Transcriptional Responses to JHIII and Ecdysone

In order to study the response of *NlGro* and *NlGro1-L* to juvenile hormone and the ecdysone signaling pathway, we used juvenile hormone and ecdysone to treat *Nilaparvata lugens* nymphs, and measured the changes in expression of *NlGro* and *NlGro1-L* by qRT-PCR. Furthermore, the expression of *NlGro* and *NlGro1-L* was measured after down-regulated the expression of juvenile hormone-related genes: *NlMet*, *NlβFtz-f1*, *NlTai*, and *NlKr-h1*, and ecdysone-related genes: *NlE93*, *NlECR*, *NlUspA*, *NlUspB*, and *NlBr*.

**Figure 3:**
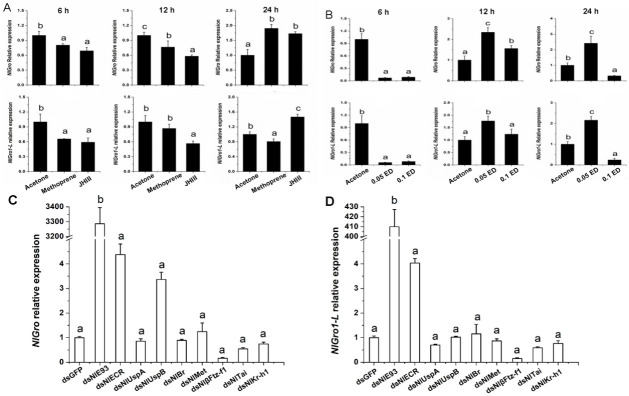
Expressions of *NlGro* and *NlGro1-L* were regulated by JH and ecdysone. (**A**) The effect of JHIIIon the expression of *NlGro* and *NlGro1-L.* Acetone: control; Methoprene: a JH analogue; JHIII: Juvenile Hormone III. (**B**) The effect of ecdysone on the expression of *NlGro* and *NlGro1-L.* Acetone: control; 0.05 ED: 0.05, ecdysone; 0.1 ED: 0.1, ecdysone. (**C**) Changes in the expression of *NlGro* after hormone-related genes are down-regulated. (**D**) Changes in the expression of *NlGro1-L* after hormone-related genes are down-regulated. One-way analysis of variance (Duncan’s method) was used. The same letter indicates that there is no significant difference, and different letters indicate a significant difference, *p* < 0.05.

When the fifth instar nymphs were treated with JHIII, the expression of *NlGro* and *NlGro1-L* was significantly reduced 6 h/12 h after treatment (Figure 3A). However, the expression of *NlGro* and *NlGro1-L* increased significantly 24 h after treatment (Figure 3A), suggesting JHIII treatment has a significant effect on the expression of *NlGro* and *NlGro1-L*. When the fifth instar nymphs were treated with ecdysone (ED), the expression of *NlGro* and *NlGro1-L* was significantly reduced 6 h after treatment (Figure 3B), while the expression of *NlGro* and *NlGro1-L* varied 12 h/24 h after treatment (Figure 3B). Again, ED treatment caused a significant change in the relative expression of *NlGro* and *NlGro1-L*.

### 3.4. Effects of Down-Regulating Hormone-Related Genes

We used RNAi to down-regulate *NlE93*, *NlECR*, *NlUspA*, *NlUspB*, *NlBr*, *NlMet*, *NlβFtz-f1*, *NlTai*, and *NlKr-h1* in the fourth instar nymphs, and collected samples 3 days after dsRNA injection. Then, the expression of *NlGro* and *NlGro1-L* was measured (*actin* was used as the reference gene). After down-regulating the expression of hormone-related genes in the fourth instar *Nilaparvata lugens* by RNAi, the changes in the expression of *NlGro* and *NlGro1-L* were similar (Figure 3C,D). After down-regulating *NlE93* and *NlECR*, the expression of *NlGro* and *NlGro1-L* increased. Surprisingly, after down-regulating *NlE93* alone, the expression of *NlGro* and *NlGro1-L* increased significantly, up to hundreds to thousands of times (Figure 3C,D). After down-regulating *NlβFtz-f1*, *NlTai* or *NlKr-h1*, the expression of *NlGro* and *NlGro1-L* decreased. After down-regulating *NlKr-h1* alone, the expression of *NlGro* and *NlGro1-L* decreased to 75%. After down-regulating *NlTai* alone, the expression of *NlGro* and *NlGro1-L* decreased by 50%. After down-regulating *NlβFtz-f1* alone, the expression of *NlGro* and *NlGro1-L* decreased to about 15% (Figure 3C,D). In summary, the expression of *NlGro* and *NlGro1-L* was more sensitive to the down-regulation of ecdysone signaling-related genes than that of juvenile hormone-related genes.

### 3.5. The Emergence Was Disrupted after Down-Regulation of NlGro and NlGro1-L

**Figure 4:**
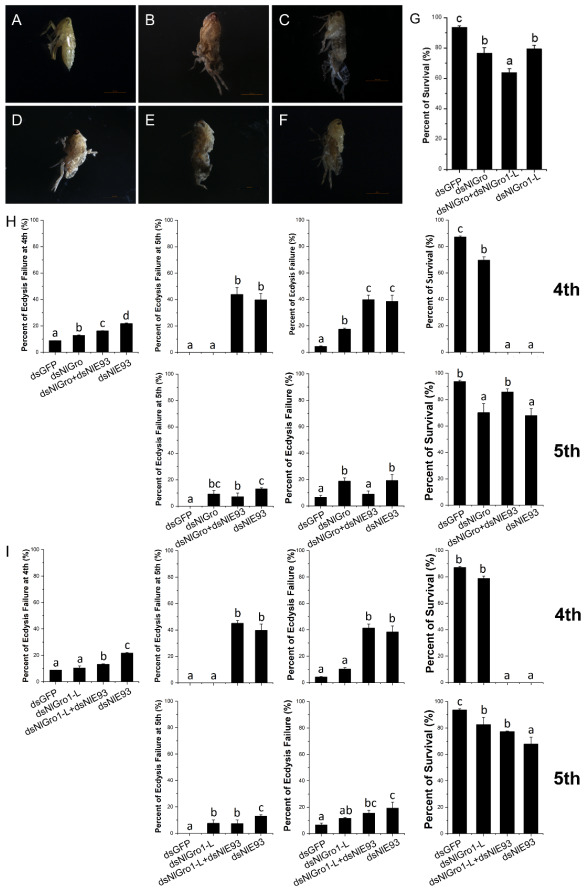
Molting disruption by down-regulating *NlGro* and *NlGro1-L*. (**A**–**F**) normal and disrupted adult emerged after injection of dsRNA: (**A**) Control: dsGFP; (**B**), dsNlGro; (**C**), dsNlGro1-L; (**D**), dsNlE93; (**E**), dsNlGro+dsN1E93; (**F**), dsNlGro1-L+dsNlE93. (**G**) Percent of survival of brown planthoppers injected with *NlGro* and *NlGro1-L*. Duncan’s multiple comparison was used. The letters a, b, and c indicate whether there is a difference in statistics: the same letter indicates that there is no difference, while different letters indicate significant differences, *p* < 0.05. (**H**) Percent of ecdysis failure and survival after injection of dsNlGro. (**I**) Percent of ecdysis failure and survival after injection of dsNlGro-L.

In order to understand the functions of *NlGro* and *NlGro1-L* during the growth, development, and reproduction, we used dsRNA to down-regulate the expression of *NlGro* and *NlGro1-L* in the nymphs, and studied molting, survival, ovarian development, and reproduction. After the injection of *NlGro* dsRNA and *NlGro1-L* dsRNA, we found the nymphs failed to emerge as adults and died during the molting process (Figure 4A–C). The emergence rate of the control is 93%, which decreased to about 80% after down-regulating *NlGro* or *NlGro1-L* alone. After down-regulating *NlGro* and *NlGro1-L* in combination, the emergence rate decreased to lower than 65% (Figure 4D,G).

The expression of *NlGro* and *NlGro1-L* increased dramatically after down-regulating *NlE93* (Figure 3G). Thus, we speculated that *NlE93* may interact with *NlGro* and *NlGro1-L*. When *NlGro* dsRNA was injected into the fourth instar nymphs, 12.87% of the fourth instar nymphs failed to molt (Figure 4H). Moreover, the emergence rate of the fifth instar nymphs (69.75%, Figure 4H) was lower than that of the control (dsGFP, 87.13%, Figure 4H). When *NlE93* was down-regulated alone, 21.73% of the fourth instar nymphs failed to molt, 39.81% of the fifth instar nymphs died, 38.46% of the fifth instar nymphs failed to molt, and no adults emerged (Figure 4H). When *NlGro* and *NlE93* were down-regulated together, no adults emerged (Figure 4D–F,H).

When dsRNA was injected into the fifth instar nymphs and *NlGro* was down-regulated alone, the mortality rate was 9.26%, the molting failure rate was 18.75%, and 71.99% of the nymphs molted to adults (Figure 4E,F,I). When *NlE93* was down-regulated, the mortality rate was 13.1%, 19.35% of the adult failed to molt, and only 67.55% succeeded in eclosion (Figure 4I). After down-regulating *NlGro* and *NlE93* together, the mortality rate and the rate of molting failure decreased. The emergence rate rose to 83.89%. This is reminiscent of the dramatic increase of *NlGro* and *NlGro1-L* after the down-regulation of *NlE93* (Figure 3G)*,* suggesting a compensation effect by down-regulation of *NlE93* (Figure 4I).

In addition, we used RNAi to down-regulate *NlGro* or *NlGro1-L* in the third, fourth, and fifth instar nymphs, collected samples 3 days after dsRNA injection, and detected RNAi efficiency by qRT-PCR. We found that the expression of each gene was significantly reduced, indicating the RNAi is successful (Figures S4 and S5).

### 3.6. Ovarian Grading and Oviposition after Down-Regulation of NlGro and NlGro1-L

**Figure 5:**
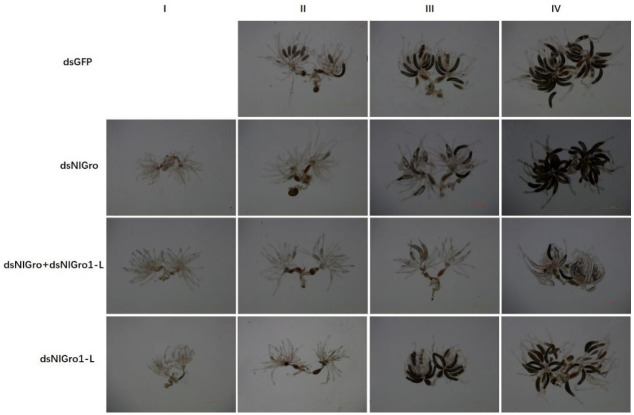
Ovarian development changed after down-regulation of *NlGro* and *NlGro1-L*. Ovarian grade (**I**) (dsNlGro, dsNlGro-L, dsNlGro + dsNlGro-L), (**II**) (dsGFP, dsNlGro, dsNlGro-L, dsNlGro + dsNlGro-L), (**III**) (dsGFP, dsNlGro, dsNlGro-L, dsNlGro + dsNlGro-L), and (**IV**) (dsGFP, dsNlGro, dsNlGro-L, dsNlGro + dsNlGro-L) are shown.

**Figure 6:**
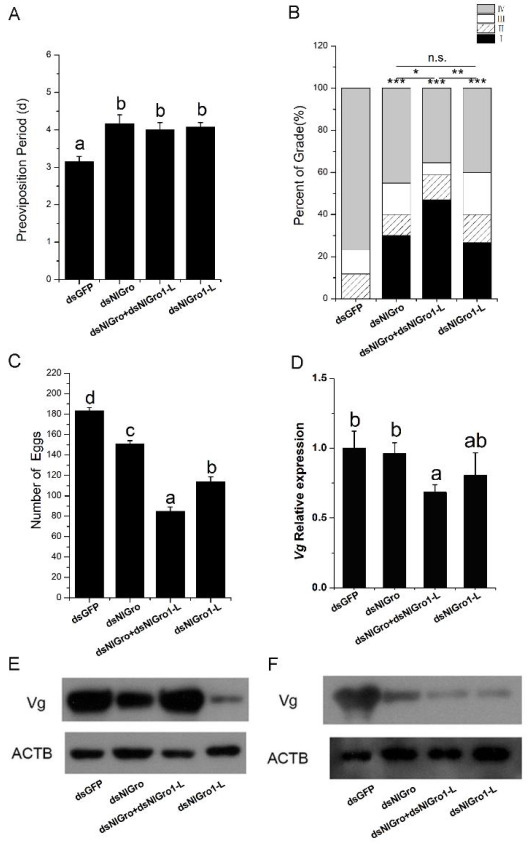
Down-regulation of *NlGro* and *NlGro1-L* affected ovary development and Vg expression. (**A**) Pre-oviposition period after down-regulation of *NlGro* and *NlGro1-L*; Duncan’s multiple comparison was used. The letters a and b indicate whether there is a statistical difference; the same letter represents no difference, while different letters represent significant differences, *p* < 0.05. (**B**) Ovarian grading after down-regulating *NlGro* and *NlGro1-L*; independent sample *t* test was used (Student’s t test), n.s.: no significant difference, * *p* < 0.05, ** *p* < 0.01, *** *p* < 0.001. (**C**) The total number of eggs reduced after down-regulating *NlGro* and *NlGro1-L*; Duncan’s multiple comparison was used. The letters a and b indicate whether there is a statistical difference; the same letter represents no difference, while different letters represent significant differences, *p* < 0.05. (**D**) Relative expression of Vg after the down-regulation of *NlGro* and *NlGro1-L*; Duncan’s multiple comparison was used. The letters a and b indicate whether there is a statistical difference; the same letter represents no difference, while different letters represent significant differences, *p* < 0.05. (**E**,**F**) Western blot detection of Vg protein after down-regulation of *NlGro* and *NlGro1-L*: (**E**) Abdomen, (**F**) Ovary.

Three days after the emergence of females, the ovaries were dissected. The ovary of the females injected with dsGFP developed into grades II, III, or IV (Figure 5). The other three treatments developed into grade I, II, III, or IV ovaries (Figure 5). The representative images of each treatment are shown in Figure 5. After *NlGro* and *NlGro1-L* were down-regulated alone or in combination, the pre-oviposition period of females was prolonged to 4 days, which is 1 day more than the control (dsGFP) (Figure 6A). Moreover, the ovarian development was delayed (Figure 6B). Compared with the control group, the number of ovaries that developed into grade IV was reduced, while the number of ovaries retained in grade I was increased, indicating that ovarian development was significantly delayed after *NlGro* or *NlGro1-L* were down-regulated (Figure 6B). After down-regulating dsNlGro and dsNlGro1-L together, the ovarian development was significantly delayed, 50% of the ovaries remained at grade I, and the number of ovaries that have developed into grades III and IV was significantly reduced (Figure 6B).

Furthermore, after *NlGro* or *NlGro1-L* was down-regulated, the number of eggs laid was significantly reduced (Figure 6C). When *NlGro* and *NlGro1-L* were down-regulated together, the number of eggs laid also reduced significantly (85) compared with that of the control (183, Figure 6C).

### 3.7. Gro and Gro1-L Regulate Vg Expression

The expression *Vg* was measured by qRT-RCR after injecting dsGFP, dsNlGro, and dsNlGro1-L (alone or in combination). The results showed that after down-regulating *NlGro* or *NlGro1-L*, the expression of *Vg* decreased but not significantly at a transcriptional level (Figure 6D). When both *NlGro* and *NlGro1-L* were down-regulated, the expression of *Vg* decreased significantly (Figure 6D).

To understand the change of Vg expression at a protein level, the abdomen (without ovary) and ovary were collected. Vg was detected by anti-Vg through western blot (Figure 6E,F). The Vg expression in the abdomen was not significantly decreased compared with the control, when *NlGro* or *NlGro1-L* was down-regulated, respectively (Figure 6E,F). However, Vg decreased significantly in the abdomen when *NlGro* and *NlGro1-L* were down-regulated together (Figure 6E,F).

## 4. Discussion

This study found that the expression profiles of *NlGro* and *NlGro1-L* are similar, and they are expressed in all tissues at all developmental stages (Figure 2). *NlGro* and *NlGro1-L* are highly expressed in the head, wings, ovary, and testis, indicating that they might also be directly involved in the development of ovaries and testes, thereby affecting the reproduction. The high expression level indicates that *NlGro* and *NlGro1-L* play an important role.

**Figure 7:**
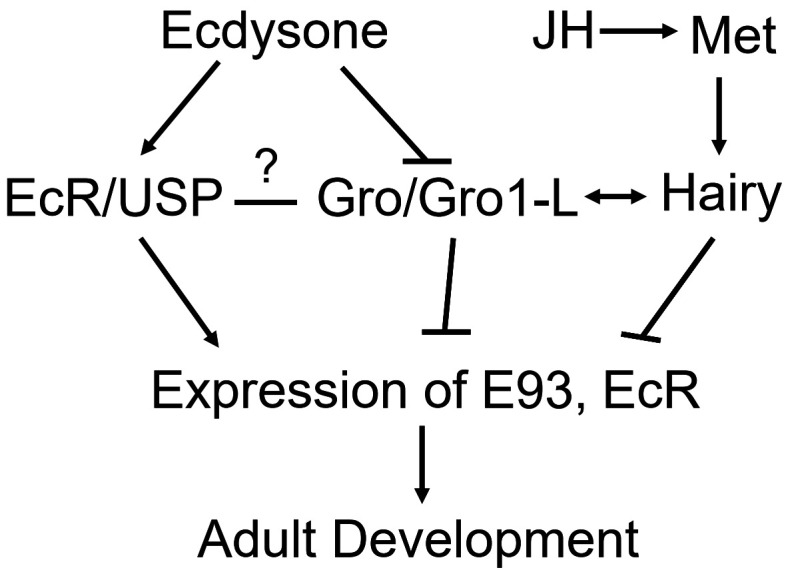
Model of *Gro* and *Gro1-L* involved in JH and the ecdysone signaling pathway.

Juvenile hormone and ecdysone interact with each other to regulate molting and reproduction [4]. JHIII can regulate changes in the expression of *NlGro* and *NlGro1-L*. After *NlβFtz-f1*, *NlTai*, and *NlKr-h1* were down-regulated, the expression of *NlGro* and *NlGro1-L* was decreased (Figure 3C,D), especially when the expression of *NlβFtz-f1* was down-regulated, while the expression of *NlGro* and *NlGro1-L* decreased to 15% (Figure 3C,D). Therefore, we speculate that the expression of *NlGro* and *NlGro1-L* is related to *NlβFtz-f1*. *βFtz-f1* is highly expressed in the molting stage of the nymphs, and it also plays a role in the formation and development of the larval cuticle [30]. βFtz-f1 regulates the formation of yolk and affects oviposition in *Aedes aegypti* [31]. It also regulates the reproduction in *Tribolium castaneum* [32]. We speculate that *NlGro* and *NlGro1-L* may affect brown planthopper molting and ovarian development through the interaction with βFtz-f1. Treatment with ecdysone (20E) or down-regulation of its receptor NlECR or key downstream transcription factor NlE93, the expression of *NlGro* and *NlGro1-L* was significantly changed (Figure 4), suggesting *NlGro* and *NlGro1-L* can participate in the growth and development at different stages. The expression of *NlGro* and *NlGro1-L* was significantly reduced after ecdysone treatment (Figure 3), suggesting the negative regulation of *NlGro* and *NlGro1-L* by ecdysone and its receptor. The expression of *NlE93* increased significantly after down-regulating *NlGro* and *NlGro1-L* (Figure 3), suggesting the negative regulation of *NlE93* by *NlGro* and *NlGro1-L* (Figure 7). It is worth noting that NlGro has both a highly conserved Q domain and WD40 domain, while *NlGro1-L* only has WD40 domain (Figure 1). Down-regulation of *NlGro* and *NlGro1-L* together disrupted the growth and development (Figure 5), delayed the ovarian development, and reduced the number of eggs laid (Figure 6), indicating that *NlGro* and *NlGro1-L* might have a synergistic effect. Moreover, a previous study has shown that Hairy and Groucho mediate the action of juvenile hormone receptor Methoprene-tolerant in gene repression [25]. We surmise Gro and Gro1-L mediate the interaction between ecdysone signaling and JH signaling (Figure 7).

Vitellogenin (Vg) is the precursor of vitellin. After the fat body synthesizes Vg protein, which is transported to the hemocyte and is taken up by the developing oocytes through receptor-mediated endocytosis, it then undergoes a series of modifications. After modification, it is deposited as yolk protein, which provides nutrition for the development of the embryo [33]. Hormone and nutritional pathways can regulate the synthesis of Vg and the uptake by oocytes, which is required for the successful reproduction of females [34,35]. In *Rhodnius prolixus*, if the expression of *Vg* is down-regulated by RNAi, egg production would be inhibited, because the nutrient transport process in *Rhodnius prolixus* is compromised [36]. In the cotton boll weevil, *Anthonomus grandis,* when *Vg* expression is down-regulated, the egg production would be reduced [37]. After the fifth instar nymphs were injected with dsNlGro and dsNlGro1-L alone or in combination, both qRT-PCR and western blot showed that the expression of Vg was reduced, and it was more dramatic when two genes were down-regulated together, indicating that the delayed development of the ovary and the decrease in egg number may be a direct result of the down-regulation of Vg.

In conclusion, this study will help us to further understand the functions of *NlGro* and *NlGro1-L*, and their molecular mechanism of action, and provides a basis for the development of more direct and effective pest management solutions in the future.

## Data Availability

Not applicable.

## Supplementary Materials

The following supporting information can be downloaded at: https://www.mdpi.com/article/10.3390/ijms23031197/s1.
