# Supplementary material for: Role of Groucho and Groucho1-like in Regulating Metamorphosis and Ovary Development in Nilaparvata lugens (Stål)
Source: Int J Mol Sci. 2022 Jan 21;23(3):1197. doi: 10.3390/ijms23031197 (PMC8835753; doi:10.3390/ijms23031197)
Supplement: Supplementary file 1 [file ijms-23-01197-s001.zip › ijms-1527664-supplementary.pdf]

Supporting Information

Table S1 Primers for Cloning

Name	Sequence (5'-3')
NlGroF	TTAACGTGTTCGTAATGTGT
NlGroR	TTAGATGTAATTTACCTGGA
NlGro1-LR	TACTCGTTCCATGTAAGCGC
NlGro1-LR	CACAGTCCTTAGGGCTTTGC

Table S2 Primers for qRT-PCR

Name	Sequence (5'-3')	Amplification efficiency
NlActinQF	TGGACTTCGAGCAGGAAATGG	99%
NlActinQR	ACGTCGCACTTCATGATCGAG	
NlGroQF	GATCTTCTCGCTGGGCTACT	96%
NlGroQR	AGCTGGTACTTGTCAGGCTT	
NlGro1-LQF	CGCAGAGAGAGCATACACG	95%
NlGro1-LQR	GTGGGCATGGAGAATAGCAA	
NlRP15QF	TAAAAATGGCAGACGAAGAAGAGCCCAA	93%
NlRP15QR	TTCCACGGTTGAAACGTCTGCG	
NlRP11QF	CCGATCGTGTGGCGTTGAAGGG	93%
NlRP11QR	ATGGCCGACATTCTTCCAGGTCC	
NlMetQF	AAAGCCGGTGTCTTTGAAGT	91%
NlMetQR	TTTCAGGATTTGGCCGTTCA	

NlβFtz-f1QF	CCATGAGAACCCGTAATCCG	95%
NlβFtz-f1QR	CACACTCGAGTCCCTTGATG	
NlTaiQF	ATGATCCCAACCACTTCAGC	93%
NlTaiQR	TTCCACTCACACTACCACCA	
NlKr-h1QF	TGATGAGGCACACGATGACT	96%
NlKr-h1QR	ATGGAAGGCCACATCAAGAG	
NlE93QF	AACAACCTCCCGAAATGCAT	91%
NlE93QR	TGCATATGATGGTGGTGGTG	
NlECRQF	AAGGCATGTTCCAGCGAAG	97%
NlECRQR	GTGTAGGGCTGGTTGTTGG	
NlUspAQF	CGAGGACTGAGCTTGGAGAA	100%
NlUspAQR	CTAAAGTTGCCCACCGTGAG	
NlUspBQF	TGCTTGTCTTCTTATCATCGCT	92%
NlUspBQR	GTCCCACCGAATTCAACGAC	
NlBrQF	CCAGGCAAACAACCCAATC	95%
NlBrQR	CTACACTGCCCCTCTTCACG	
NlVgQF	CACTGCCCGTGCTGTGCTCTA	91%
NlVgQRNlBrQR	TGACTTCCTTGCTTTGCTCCC	

Table S3 Primers for dsRNA Synthesis

Name	Sequence (5'-3')
ds*GFP*F	GGATCCTAATACGACTCACTATAGGAAGGGCGAGGAGCTGTTCACCG
ds*GFP*R	GGATCCTAATACGACTCACTATAGGCAGCAGGACCATGTGATCGCGC
ds*NlGro*F	TAATACGACTCACTATAGGGAGACCACCAATCACCTTCTCCGATCCG
ds*NlGro*R	TAATACGACTCACTATAGGGAGACCACCTGCAAGGCGATTAAGTTGG
ds*NlGro1-L*F	TAATACGACTCACTATAGGGAGACCACGCTTGTGCGACAGTTCCAAG
ds*NlGro1-L*R	TAATACGACTCACTATAGGGAGACCACTAGATTTGCTTCCGCGTGGT
ds*NlMet*F	TAATACGACTCACTATAGGGAGACCACCAACCAGCAGATGAACCTGA
ds*NlMet*R	TAATACGACTCACTATAGGGAGACCACGCAAAGCCTCGTACTCTTGG
ds*Nlβftz-f1*F	TAATACGACTCACTATAGGGAGACCACCGACCAGATCTCGTTGCTGA
ds*Nlβftz-f1*R	TAATACGACTCACTATAGGGAGACCACGCAGCCACAAGTAGAATCCG
ds*NlTai*F	TAATACGACTCACTATAGGGAGACCACTTCATTCATTCAGGCTCGGC
ds*NlTai*R	TAATACGACTCACTATAGGGAGACCACCCACTCACACTACCACCACT
ds*NlKr-h1*F	TAATACGACTCACTATAGGGAGACCACGTGGGGTTCAGTCCTGAGGA
ds*NlKr-h1*R	TAATACGACTCACTATAGGGAGACCACCAGTCGAACACACACCGGAG
ds*NlE93*F	TAATACGACTCACTATAGGGAGACCACGCCAGCTTACATGACGAAGA
ds*NlE93*R	TAATACGACTCACTATAGGGAGACCACCAGAGTGCAGGATAAATGAC
ds*NlECR*F	TAATACGACTCACTATAGGGAGACCACCTTCGGTTGGTGGGTCTCTC
ds*NlECR*R	TAATACGACTCACTATAGGGAGACCACGCATTGTCCACCTTCATGCG
ds*NlUspA*F	TAATACGACTCACTATAGGGAGACCACTCGGTGGTGCTCTTTTGGTG
ds*NlUspA*R	TAATACGACTCACTATAGGGAGACCACAAGTGTGGTGATCTACTGGTCA

ds*NlUspB*F	TAATACGACTCACTATAGGGAGACCACTGCTTGTCTTCTTATCATCGCT
ds*NlUspB*R	TAATACGACTCACTATAGGGAGACCACGTAAGTGTGGTGATCTACTGG
ds*NlBr*F	TAATACGACTCACTATAGGGAGACCACCGTCATCTCGGACAGTGCTA
ds*NlBr*R	TAATACGACTCACTATAGGGAGACCACCGAAGTCCCTGAGACAAAGC

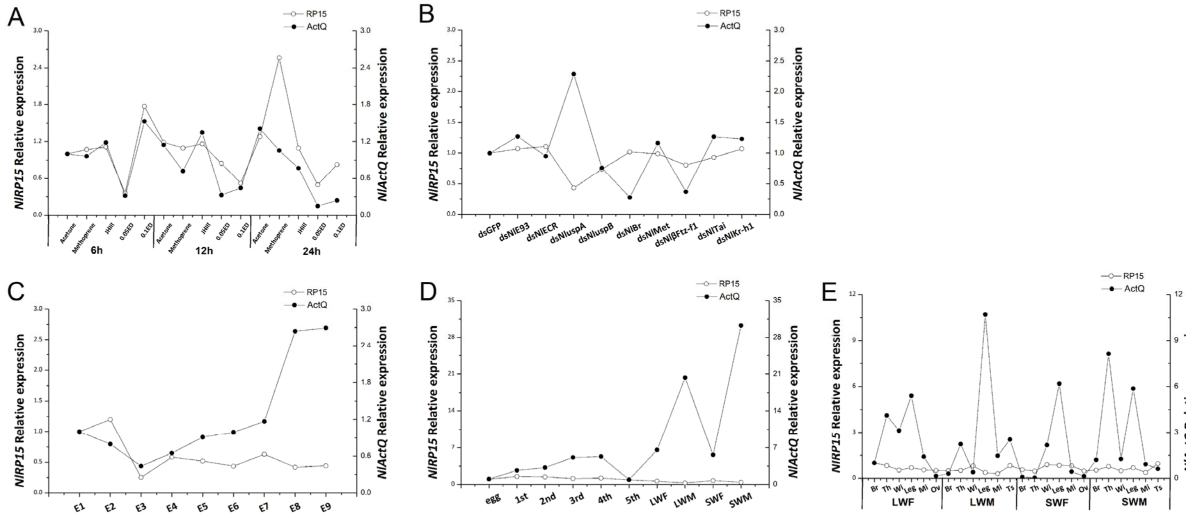

Figure S1 Stability of the reference genes, *NlRP15* and *NlActin*.

Here *NlRP11* was used as a reference gene. A-E are different groups of templates. A, JHIII and ecdysone treatment experiment; B, RNAi experiment; C, samples from different embryonic stages, E: Embryo; 1-9 day(s) after eggs were laid; D, developmental stages. Egg: egg; 1st to 5th: 1st to 5th instar nymph; LWF: long-winged female, LWM: long-winged male, SWF: short-winged female, SWM: short-winged male; E, different tissues. Br: Brain; Th: Thorax; Wi: fore-wing; Leg: Leg; Mi: mid-gut; Ov: Ovary; Ts: testis; LWF: long-winged female, LWM: long-winged male, SWF: short-winged female, SWM: short-winged male.

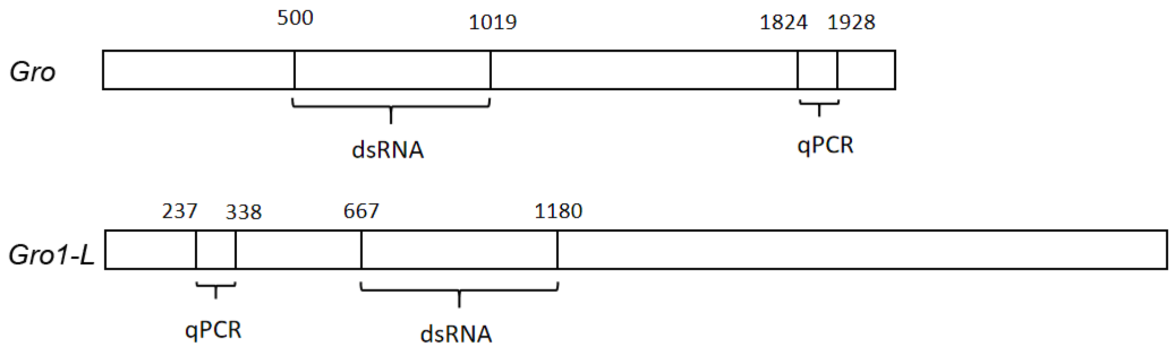

Figure S2 Region of dsRNA and qRT-PCR primers.

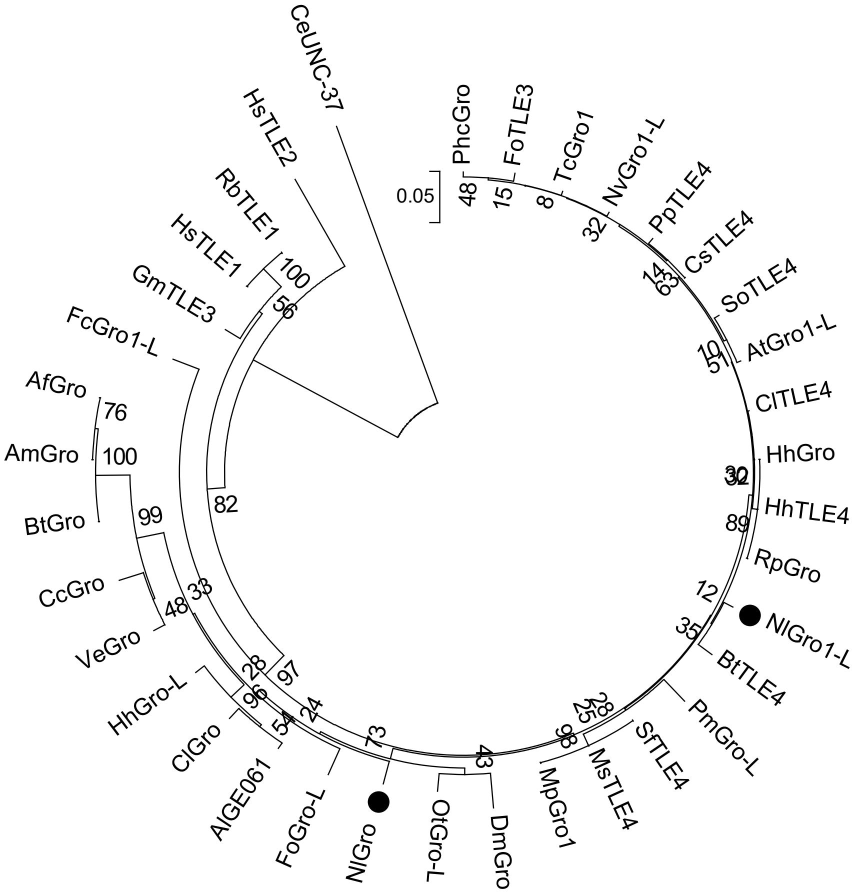

Figure S3 Phylogenic analysis of Gro and Gro1-L

NlGro, *Nilaparvata lugens Gro* (XP_022195835.1); *NlGro1-L*, *Nilaparvata lugens Gro1-L* (XP_022189353.1); *DmGro*, *Drosophila melanogaster Gro* (NP_733133.1); *HsTLE1*, *Homo sapiens TLE1* (NP_001290032.1); *HsTLE2*, *Homo sapiens TLE2* (NP_003251.2); *CeUNC-37*, *Caenorhabditis elegans UNC-37* (NP_491932.1); *AlGE061*, *Apolygus lucorum GE061*

(KAE9440368.1); *HhGro-L*, *Halyomorpha halys Gro-L* (XP_014275615.1); *ClGro*, *Cimex lectularius Gro* (XP_024082808.1); *FoGro-L*, *Frankliniella occidentalis Gro-L* (XP_026282736.1); *VeGro*, *Vollenhovia emeryi Gro* (XP_011882917.1); *CcGro*, *Cephus cinctus Gro* (XP_024938868.1); *BtGro*, *Bombus terrestris Gro* (XP_012164601.1); *AmGro*, *Apis mellifera Gro* (XP_006564194.1); *AfGro*, *Apis florea Gro* (XP_012342289.1); *FcGro1-L*, *Folsomia candida Gro1-L* (XP_021968336.1); *GmTLE3*, *Gadus morhua TLE3* (XP_030232419.1); *RbTLE1*, *Rhinopithecus bieti TLE1* (XP_017727850.1); *HhGro*, *Halyomorpha halys Gro* (XP_014282834.1); *ClTLE4*, *Cimex lectularius TLE4* (XP_014258830.1); *HhTLE4*, *Halyomorpha halys TLE4* (XP_014282837.1); *RpGro*, *Riptortus pedestris Gro* (BAN21009.1); *SoTLE4*, *Sitophilus oryzae TLE4* (XP_030760956.1); *PpTLE4*, *Photinus pyralis TLE4* (XP_031338821.1); *TcGro1*, *Tribolium castaneum Gro1* (XP_008201485.1); *NvGro1-L*, *Nicrophorus vespilloides Gro1-L* (XP_017770757.1); *BtTLE4*, *Bemisia tabaci TLE4* (XP_018908679.1); *AtGro1-L*, *Aethina tumida Gro1-L* (XP_019871485.1); *SfTLE4*, *Sipha flava TLE4* (XP_025405291.1); *MsTLE4*, *Melanaphis sacchari TLE4* (XP_025193209.1);*FoTLE3*, *Frankliniella occidentalis TLE3* (XP_026289757.1); *MpGro1*, *Myzus persicae Gro1* (XP_022168813.1); *CsTLE4*, *Cryptotermes secundus TLE4* (PNF31698.1); *PmGro-L*, *Papilio machaon Gro-L* (XP_014357517.1); *PhcGro*, *Pediculus humanus corporis Gro-L* (XP_002427252.1); *OtGro-L*, *Onthophagus taurus Gro-L* (XP_022914630.1). The phylogenetic tree was constructed by Neighbor-joining method using the MEGA 6 software. Bootstrap=1000

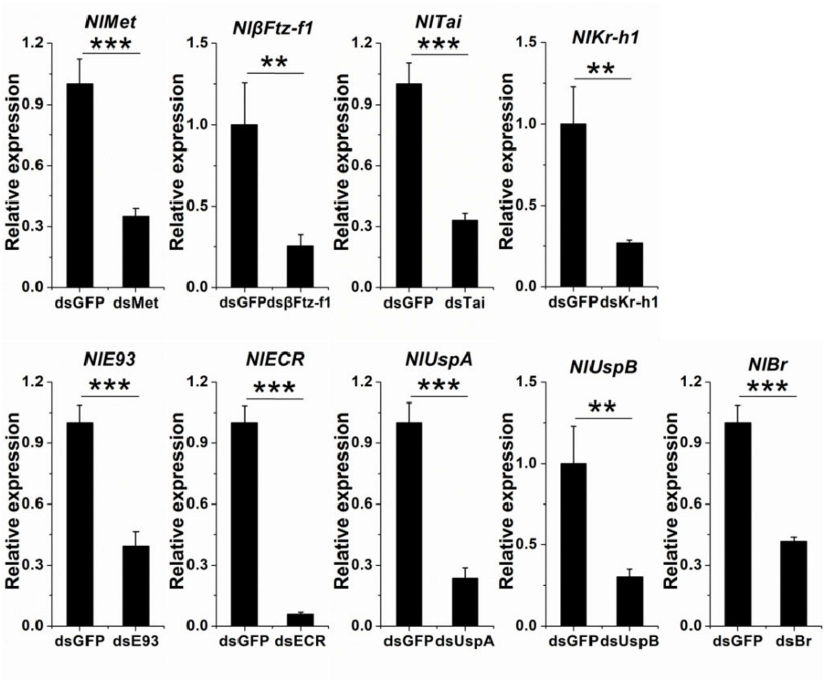

Figure S4 RNAi efficiency of hormone related genes

Student's *t* test was used. **：*P*<0.01，***：*P*<0.001.

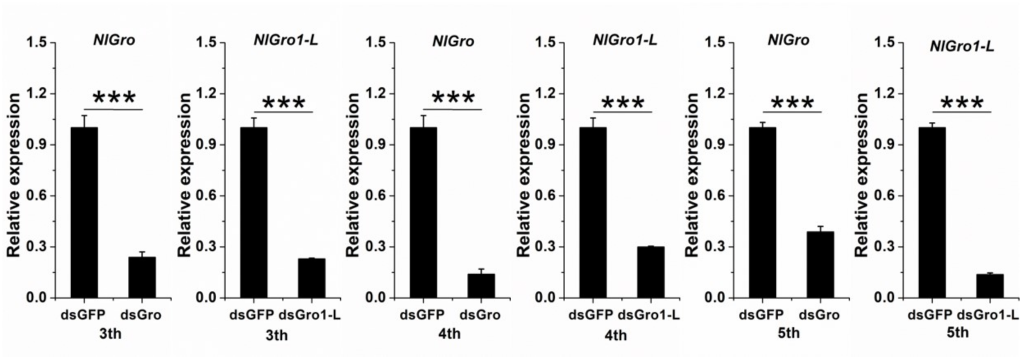

Figure S5 RNAi efficiency of *NlGro* and *NlGro1-L*

Student's *t* test was used. ***：*P*<0.001.
